# Limits of [^18^F]-FLT PET as a Biomarker of Proliferation in Oncology

**DOI:** 10.1371/journal.pone.0058938

**Published:** 2013-03-15

**Authors:** Eliot T. McKinley, Gregory D. Ayers, R. Adam Smith, Samir A. Saleh, Ping Zhao, Mary Kay Washington, Robert J. Coffey, H. Charles Manning

**Affiliations:** 1 Vanderbilt University Institute of Imaging Science (VUIIS), Vanderbilt University Medical Center, Nashville, Tennessee, United States of America; 2 Department of Biomedical Engineering, Vanderbilt University, Nashville, Tennessee, United States of America; 3 Department of Biostatistics, Vanderbilt University Medical Center, Nashville, Tennessee, United States of America; 4 Department of Pathology, Vanderbilt University Medical Center, Nashville, Tennessee, United States of America; 5 Department of Medicine, Vanderbilt University School of Medicine, Nashville, Tennessee, United States of America; 6 Vanderbilt Ingram Cancer Center, Vanderbilt University Medical Center, Nashville, Tennessee, United States of America; 7 Department of Radiology and Radiological Science, Vanderbilt University Medical Center, Nashville, Tennessee, United States of America; 8 Program in Chemical and Physical Biology, Vanderbilt University Medical Center, Nashville, Tennessee, United States of America; 9 Department of Neurosurgery, Vanderbilt University Medical Center, Nashville, Tennessee, United States of America; The University of Kansas Medical Center, United States of America

## Abstract

**Background:**

Non-invasive imaging biomarkers of cellular proliferation hold great promise for quantifying response to personalized medicine in oncology. An emerging approach to assess tumor proliferation utilizes the positron emission tomography (PET) tracer 3’-deoxy-3’[^18^F]-fluorothymidine, [^18^F]-FLT. Though several studies have associated serial changes in [^18^F]-FLT-PET with elements of therapeutic response, the degree to which [^18^F]-FLT-PET quantitatively reflects proliferative index has been continuously debated for more that a decade. The goal of this study was to elucidate quantitative relationships between [^18^F]-FLT-PET and cellular metrics of proliferation in treatment naïve human cell line xenografts commonly employed in cancer research.

**Methods and Findings:**

[^18^F]-FLT-PET was conducted in human cancer xenograft-bearing mice. Quantitative relationships between PET, thymidine kinase 1 (TK1) protein levels and immunostaining for proliferation markers (Ki67, TK1, PCNA) were evaluated using imaging-matched tumor specimens. Overall, we determined that [^18^F]-FLT-PET reflects TK1 protein levels, yet the cell cycle specificity of TK1 expression and the extent to which tumors utilize thymidine salvage for DNA synthesis decouple [^18^F]-FLT-PET data from standard estimates of proliferative index.

**Conclusions:**

Our findings illustrate that [^18^F]-FLT-PET reflects tumor proliferation as a function of thymidine salvage pathway utilization. Unlike more general proliferation markers, such as Ki67, [^18^F]-FLT PET reflects proliferative indices to variable and potentially unreliable extents. [^18^F]-FLT-PET cannot discriminate moderately proliferative, thymidine salvage-driven tumors from those of high proliferative index that rely primarily upon *de novo* thymidine synthesis. Accordingly, the magnitude of [^18^F]-FLT uptake should not be considered a surrogate of proliferative index. These data rationalize the diversity of [^18^F]-FLT-PET correlative results previously reported and suggest future best-practices when [^18^F]-FLT-PET is employed in oncology.

## Introduction

**Figure 1:**
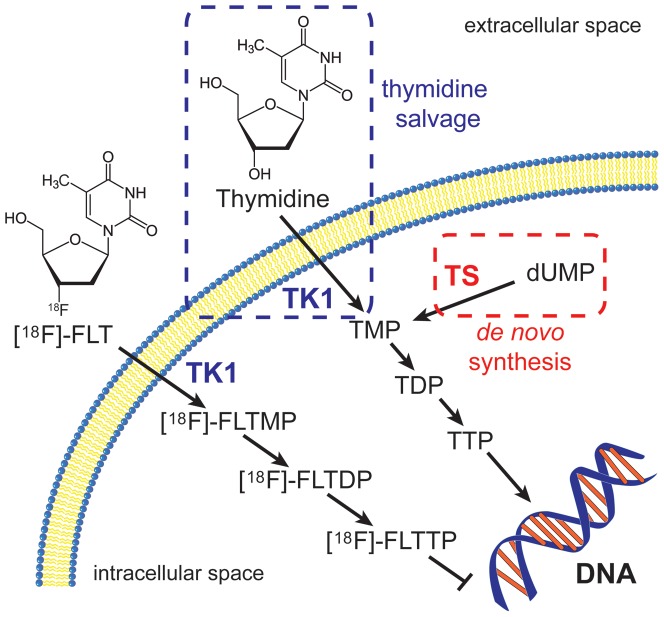
Thymidine salvage and *de novo* synthesis pathways. In thymidine salvage, thymidine is transported across the cell membrane and phosphorylated by TK1 into thymidine monophosphate (TMP). The thymidine is further phosphorylated into thymidine diphosphate (TDP) and thymidine triphosphate (TTP) and then incorporated into DNA. Alternatively, using the *de novo* synthesis pathway, deoxyuridine monophosphate (dUMP) is converted to TMP by TS which can then be further phosphorylated and incorporated into DNA. Similarly to thymidine, [^18^F]-FLT is transported into the cell and phosphorylated into [^18^F]-FLT monophosphate ([^18^F]-FLTMP) and trapped by TK1. [^18^F]-FLTMP can be further phosphorylated into [^18^F]-FLT diphosphate ([^18^F]-FLTDP) and [^18^F]-FLT triphosphate ([^18^F]-FLTTP), however, due to the substitution of OH with ^18^F in the 5-prime position, [^18^F]-FLTTP is not incorporated into the DNA.

Non-invasive molecular imaging biomarkers of cellular proliferation hold great promise for characterizing tumors and predicting their response to personalized therapeutic regimens. To this end, positron emission tomography (PET) tracers based upon precursors for DNA synthesis have been explored and include 11-carbon ([^11^C]) and 18-fluorine ([^18^F]) labeled nucleosides and related structural analogues [1], [2], [3], [4]. The most promising and widely explored of these agents has been 3’-deoxy-3’[18F]-fluorothymidine ([^18^F]-FLT) [5], [6], [7], [8]. [^18^F]-FLT-PET, serves as a surrogate of proliferation by targeting the activity of thymidine salvage, one of two distinct mechanisms that supply DNA precursors to dividing cells (**Fig. 1**).

In the salvage pathway, nucleosides including [^18^F]-FLT are transported across the cell membrane by facilitated diffusion via low-affinity, non-concentrative nucleoside carrier proteins that are conserved across nearly all animal cells [9]. Upon internalization, [^18^F]-FLT is monophosphorylated in a reaction catalyzed by the cytosolic enzyme thymidine kinase 1 (TK1). Unlike thymidine, which is further phosphorylated and subsequently incorporated into DNA, monophosphorylation of [^18^F]-FLT results in intracellular trapping and accumulation without DNA incorporation. In many tissues, TK1 activity is regulated at transcriptional, translational, and post-translational levels [9] and activity is closely correlated with the DNA synthesis phase of proliferating cells (typically late G1-S). TK1 activity is diminished in quiescent, non-proliferating cells. Many preclinical and clinical studies have been published since the late 1990s which explored [^18^F]-FLT-PET imaging to assess proliferation in various species, tumor types, and organ sites. Among these, varying degrees of correlation between [^18^F]-FLT uptake and histological markers of proliferation, such as Ki67 labeling indices [8], [10], [11], [12], [13], [14], [15], [16], have been observed. Accordingly, quantitative relationships between [^18^F]-FLT uptake and cellular proliferation in tumors have remained poorly defined.

A key factor limiting [^18^F]-FLT-PET is *de novo* thymidine pathway utilization, although the extent of this limitation is not fully appreciated. The *de novo* pathway is complementary to thymidine salvage and is fully capable of providing all the thymidine needed for DNA synthesis [9]. Through the action of the enzyme thymidylate synthase (TS), deoxyuridine monophosphate is converted to thymidine monophosphate, which is subsequently incorporated into DNA. It is widely assumed that [^18^F]-FLT PET may underestimate proliferation in *de novo* pathway-dependent tumors, yet conclusive studies determining the extent of this discrepancy have not been reported.

In this study, we show that [^18^F]-FLT-PET directly measures TK1 levels and correlates with thymidine salvage pathway utilization. We further illustrate that [^18^F]-FLT-PET poorly reflects proliferative index in tumors that utilize the *de novo* pathway. Consequently, [^18^F]-FLT-PET only correlates with proliferative index as a function of salvage pathway utilization. These data explain, in part, the diversity of [^18^F]-FLT-PET correlative results previously reported and suggest future best-practices when [^18^F]-FLT-PET is employed in oncology.

## Materials and Methods

### Cell Lines and Mouse Models

All studies were approved by the Vanderbilt University Institutional Animal Care and Use Committee and all efforts were made to minimize animal suffering. Human colorectal cancer cell lines DiFi, HCT-116, HCT-116*p21^-/-^*, HCT-116*p53^-/-^*, SW620, HT-29, and Lim2405 and the human breast cancer cell line BT474 were grown in Dulbecco’s modified Eagle’s medium, DMEM, and Colo205 grown in RPMI (Cellgro, Manassas, VA) with 10% fetal bovine serum, (Atlanta Biologicals, Lawrenceville, GA), 1% penicillin and streptomycin (Life Technologies, Grand Island, NY) at 37°C and 5% CO_2_. HCT-116, SW620, HT-29, and BT474 cells were obtained from ATCC. DiFi cells were a gift from from Dr. Bruce Boman [17], HCT-116*p21^-/-^* [18] and HCT-116*p53^-/^* ^-^ [19] cells were obtained from Dr. Bert Vogelstein’s laboratory, and Lim2405 [20] cells were obtained from Dr. Robert Whitehead (Ludwig Institute for Cancer Research). Cell line xenografts were generated in athymic nude mice (Harlan, Indianapolis, IN) as described [21] and imaged when volume reached approximately 250 mm^3^.

### [^18^F]-FLT Radiosynthesis

[^18^F]-FLT was prepared in a two-step, one-pot reaction as described [6], [22]. [^18^F]-FLT was obtained with average radiochemical purity of 98.3% and specific activity ≥ 345.5 TBq/mmol.

### Small-Animal PET Imaging

Small-animal PET imaging was performed using a dedicated Concorde Microsystems Focus 220 microPET scanner (Siemens Preclinical Solutions, Knoxville, TN). Mice were maintained under 2% isofluorane anesthesia in 100% oxygen at 2 L/min and kept warm via a circulating water heating pad for the duration of the PET scan. Animals were administered 7.4–9.3 MBq (200-250 µCi) of [^18^F]-FLT intravenously. For static scans, animals were allowed free access to food and water during a 40 minute uptake period, followed by anesthetization and a 20 minute image acquisition. Sixty minute dynamic acquisitions were initiated at the time of [^18^F]-FLT injection. PET data were reconstructed using a three-dimensional (3D) ordered subset expectation maximization/maximum a posteriori (OSEM3D/MAP) algorithm. Dynamic data was binned into twelve 5 s (0-1 min) and fifty-nine 60 s (2–60 mins) frames. The resulting three-dimensional reconstructions had an x–y voxel size of 0.474 mm and inter-slice distance of 0.796 mm.

### Image Analysis and Compartmental Modeling

ASIPro software (Siemens Preclinical Solutions) was used to manually draw three-dimensional regions of interest in the tumor volume and, for dynamic scans, the left ventricle. For static scans, [^18^F]-FLT uptake was quantified as the percentage of the injected dose per gram of tissue (%ID/g) by dividing the ROI activity by the injected dose and multiplying by 100. For dynamic scans, a 3-compartment, 4-rate-constant model was used to characterize [^18^F]-FLT pharmacokinetics as extensively described by Muzi et al [23]. The compartmental model was implemented using the Matlab-based COMKAT [24] software package. Rate constants for [^18^F]-FLT influx (K_1_) and efflux (k_2_) from the vascular compartment to the tissue compartment as well rate constants for [^18^F]-FLT phosphorylation (k_3_) and dephosphorylation (k_4_) were determined from the model. The metabolic flux of [^18^F]-FLT was calculated according to:

K_FLT_ = (K_1_ × k_3_)/(k_2_ + k_3_). (Eq. 1)

Parametric maps illustrating K_1_, K_FLT_, and %ID/g were obtained through voxel-wise compartmental modeling using the PMOD 2.6 software package (PMOD Technologies, Zurich, Switzerland).

### Immunohistochemistry (IHC)

Animals were sacrificed by cervical dislocation under isofluorane anesthesia and tumor samples were collected immediately following [^18^F]-FLT-PET, then subsequently fixed in 10% formalin for 24 h. Tissues were then transferred to 70% ethanol prior to paraffin embedding. Tissues were sectioned (4 µm thickness) and stained for proliferation markers Ki67 (#M7240, Dako, Carpinteria, CA, 1∶100 primary dilution), Proliferating Cell Nuclear Antigen (PCNA)(#V1006, Biomeda, Foster City, CA, 1∶800 primary dilution), and TK1 (#57757, Abcam, Cambridge, MA, 1∶100 primary dilution). Briefly, the tissue samples were de-paraffinized, rehydrated, and antigen retrieval was performed using citrate buffer (ph 6.0) solution for 15 minutes at 105°C followed by a 10 minute bench cool down. The samples were then treated with 3% hydrogen peroxide to eliminate endogenous peroxidase activity. The sections were subsequently blocked with a serum-free protein blocking reagent for 20 minutes. Primary antibody detection was accomplished using the following system: The tissue sections were incubated at room temperature for 60 minutes at the noted dilutions followed by a 30 minute incubation utilizing the Envision + System-HRP Labeled Polymer detection method. (Dako, Carpinteria, CA). Staining was completed after incubation with a 3,3'-Diaminobenzidine substrate-chromogen solution. Tissue slides were imaged at 40x magnification and manually scored to determine the percentage of positive cells per high power field. Tissues were evaluated by a certified GI pathologist (MKW). Three high power fields were acquired from a minimum of three separate tumors from each cell line xenograft model.

### Immunoblotting

Tumor samples were immediately collected following [^18^F]-FLT-PET and flash frozen in liquid nitrogen. Subsequently, tumor samples were homogenized and diluted to 1 µg/µl in CellLytic lysis buffer (Sigma Aldrich, St. Louis, MO). Prior to resolution by electrophoresis, 20-40 µg of protein from each sample was loaded into 7.5-12% SDS PAGE gels and transferred to PVDF membranes (PerkinElmer, Waltham, MA). Membranes were blocked overnight at 4°C in tris-buffered saline 0.1% Tween-20 (TBST) containing 5% w/v nonfat dry milk powder. Subsequently, membranes were interrogated with antibodies to TS, p21, β-actin (#5499, #2947, #4970, Cell Signaling Technologies, Danvers, MA), and TK1 (Abcam, #57757). Membranes were probed for 1 h at room temperature in TBST with 3% bovine serum albumin. Membranes were subsequently incubated for 1 h at room temperature with horseradish peroxidase-conjugated secondary antibody (Jackson ImmunoResearch, West Grove, PA) diluted 1:5000 in TBST containing 3% BSA. Western Lightning^TM^ Plus-ECL (PerkinElmer) was used for chemiluminescent detection on a Xenogen IVIS 200. Densitometry was conducted using Living Image 3.2 software (PerkinElmer).

### Cell Cycle Assay

HCT-116 and HCT-116*p21^-/-^* cells were propagated to 50% confluency in 6cm plates. Plates were washed with 2 mL PBS. Cells were then removed with trypsin/EDTA, pelleted, and fixed with 70% ethanol. Fixed cells were pelleted and resuspended in 1 mL PBS prior to the addition of 10 µL of 2 mg/mL DNase-free RNase A. The suspension was incubated at room temperature for 30 min and labeled with propidium iodide (PI; Sigma Aldrich) according to the manufacturer’s protocol. PI-stained cells were analyzed by flow cytometry (FACStar PLUS, BD, Franklin Lakes, NJ). Data analysis was performed using CellQuest software (BD) by manually gating to define and quantify sub-G0, G1, S, and G2/M populations.

### Liquid Chromatography/Mass Spectrometry (LC/MS) analysis

Endogenous thymidine levels in HCT-116 and HCT-116*p21^-/-^* xenograft tumors were analyzed similarly as described by Li et al. [25] using a TSQ Quantum Triple Quadrupole Mass Spectrometer (Thermo Scientific, Maltham, MA). Homogenized tumors were precipitated in methanol and the supernatant was injected onto a 150×2.1 mm Hypersil GOLD C18 column (Thermo Scientific) and eluted with a linear gradient of 0.1% formic acid: 0.1% formic acid in acetonitrile form 98:2 to 95:5 for more than 5 minutes at a 0.3 mL/min flow rate.

### Statistical Analysis

Differences in the distributions of [^18^F]-FLT uptake (%ID/g) and histology among cell lines were tested using the non-parametric Wilcoxon Rank Sum (Mann-Whitney U) or Kruskal-Wallis one-way analysis of variance tests using the GraphPad Prism 4 software package. Differences were considered statistically significant if p < 0.05. Non-parametric (Spearman) correlations were calculated among the mean expression values from the cell lines for each of 3 metrics of proliferation ([^18^F]-FLT %ID/g, Ki67 IHC, TK1 IHC). To assess the precision of the observed correlations, 10,000 bootstrap samples were generated from each of the independent data sets as described [26] and their resulting means and Spearman correlations were calculated. The approximate 95% confidence intervals were obtained from the 2.5^th^ and 95.5^th^ percentiles of the 10,000 bootstrap correlations. A confidence interval containing zero was not considered statistically significant (p > 0.05).

## Results

### Comparison of proliferation markers in HCT-116 and DiFi xenograft tumors

**Figure 2:**
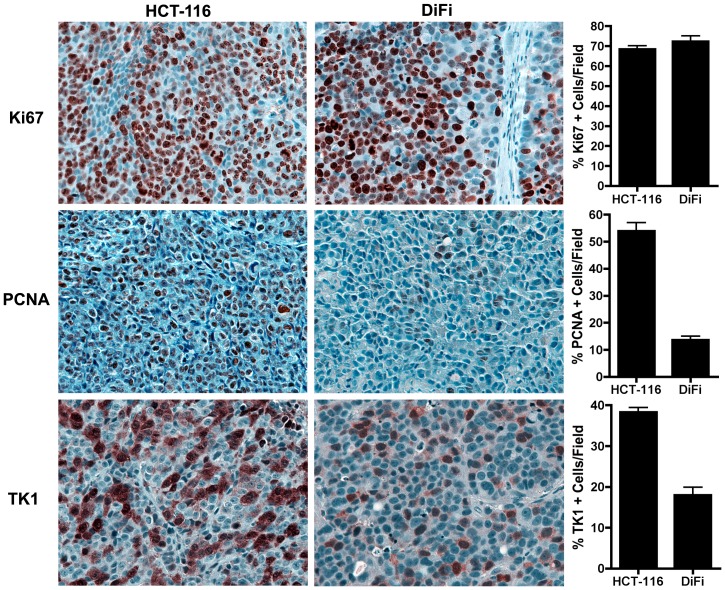
IHC markers of proliferation in HCT-116 and DiFi human CRC xenografts. Representative high power microscopic images (40x) of HCT-116 and DiFi xenograft tissue stained for Ki67, PCNA, and TK1 are shown along with quantification of the percentage of positive cells per field for each marker. HCT-116 and DiFi xenograft tissues exhibit a similar proportion of proliferating cells as measured by Ki67 indices (68.50 ± 5.52 % vs. 72.37 ± 12.64%; p  =  0.0651). The proportion of PCNA positive cells, representing S-phase, was significantly increased in HCT-116 xenografts (53.91 ± 3.18 %) compared to DiFi xenografts (13.59 ±1.48%; p  =  0.0007). Similar to PCNA, the percentage of TK1-positive cells was significantly higher in HCT-116 xenografts (38.32 ± 1.90 %) than DiFi cells (17.95 ± 3.95 %; p  =  0.0007). TK1 indices for both cell lines were reduced compared to Ki67 indices.

To understand the cellular proliferation profiles of DiFi and HCT-116 xenograft tumors, we used IHC to evaluate relationships between TK1 levels, PCNA, a marker of cells in S-phase, and Ki67, a marker of cells in any non-G0-phase (**Fig. 2**). Both xenograft models exhibited near-identical Ki67 indices, indicating a similar fraction of proliferating cells at collection (∼70% positive cells/field, p  =  0.0651). Though Ki67 was similar between the two models, interestingly, PCNA was dramatically different. In HCT-116 xenografts, we observed only slightly fewer PCNA-positive cells than Ki67-positive cells (53.91 ± 3.18 % vs. 68.50 ± 5.52 %, p  =  0.0003), yet only a small fraction of tumor cells in DiFi xenografts were PCNA-positive (13.59 ± 1.48 % vs. 72.37 ± 12.64 %, p  =  0.0003). Given its S-phase specificity, TK1 levels tracked more closely with PCNA than Ki67 and, accordingly, were significantly higher in HCT-116 tumors than DiFi tumors (p  =  0.0005). Overall, TK1 indices were significantly lower than analogous Ki67 indices in both models (HCT-116: 38.32 ± 1.90 % vs. 68.50 ± 5.52 %, p  =  0.013; DiFi: 17.95 ± 3.95 % vs. 72.37 ± 12.64 %, p  =  0.002), implying that only subset of Ki67-positive cells in either model would be expected to also accumulate [^18^F]-FLT.

### Quantitative [^18^F]-FLT PET imaging of HCT-116 and DiFi xenograft-bearing mice

**Figure 3:**
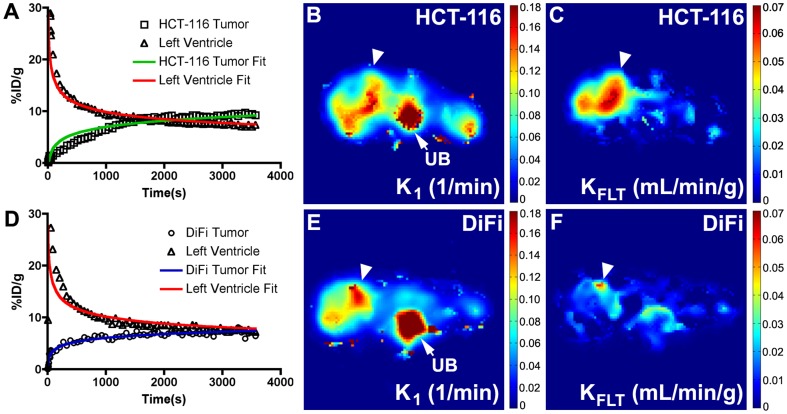
HCT-116 xenografts exhibit higher [^18^F]-FLT flux than DiFi xenografts. Representative time activity curves for HCT-116 (A) and DiFi (D) xenografts and left ventricle estimates of blood pool activity are shown. In both models, a sharp rise in activity in the blood pool is observed following tracer injection, followed by rapid clearance. Fit lines derived from compartmental modeling closely matched the measured data. Parametric maps reveal heterogeneous intratumoral delivery of [^18^F]-FLT in both HCT-116 (B) and DiFi (E) xenografts (tumor localized by arrowhead). Increased [^18^F]-FLT delivery was observed in the urinary bladder (denoted by UB) in both cell line xenografts. The tumor regions exhibiting the greatest [^18^F]-FLT flux in HCT-116 tumors (C) also exhibited the highest tracer delivery. Only modest [^18^F]-FLT flux was observed in DiFi xenografts (F), despite having similar delivery as HCT-116 xenografts (B).

We subsequently evaluated [^18^F]-FLT PET in HCT-116 and DiFi xenograft-bearing mice (**Fig. 3**). Compartmental modeling of dynamic PET imaging data was used to determine rate constants that quantitatively reflect tracer delivery, retention, and clearance. Representative time activity curves for HCT-116 and DiFi xenografts and left ventricle estimates of blood pool activity are shown (**Fig. 3A** **/2D**). Blood pool characteristics of [^18^F]-FLT were similar in both models, where the tracer demonstrated a sharp rise following intravenous injection and rapid clearance. HCT-116 xenografts accumulated [^18^F]-FLT faster and to a greater extent overall compared to DiFi xenografts. Time activity curves could be fit to a three compartment, four-parameter model in both xenograft models. Rate constants K_1_, k_2_, and k_4_, corresponding to tracer delivery, efflux, and de-phosphorylation, respectively, were not different between HCT-116 and DiFi tumors (**Table 1**). As expected based on TK1 IHC, rate constants for [^18^F]-FLT phosphorylation (k_3_) and [^18^F]-FLT flux (K_FLT_) were elevated in HCT-116 xenografts compared to DiFi. In fact, the two-fold difference in K_FLT_ between HCT-116 and DiFi xenografts was in close agreement with the difference in TK1 indices by IHC. Delivery of [^18^F]-FLT, as estimated by K_1_ values, was essentially equivalent between HCT-116 and DiFi xenografts. Parametric mapping was used to explore tracer kinetics on a voxel-by-voxel basis (**Fig. 3** **B/C; E/F**). Intratumoral delivery was heterogeneous in both tumor types (**Fig. 3B** **/E**), with evidence of modest central necrosis in both models. Elevated delivery was noted in xenograft tissue compared with adjacent non-tumor tissue, with the exception of the urinary bladder. In HCT-116 xenografts, tumor regions exhibiting the greatest [^18^F]-FLT flux also exhibited the greatest tracer delivery (**Fig. 3C**). Importantly however, parametric analysis clearly illustrated that the modest flux observed in DiFi xenografts (**Fig. 3F**) was not the result of impaired delivery, as DiFi xenografts exhibited similar tracer delivery compared with HCT-116 xenografts (**Fig. 3E** **,** **Table 1**).

**Table 1:** Kinetic parameters derived from compartmental modeling of dynamic [^18^F]-FLT PET scans in HCT-116 and DiFi tumor xenografts.

Cell Line	K_1_ (mL/min/g)	k_2_ (1/min)	k_3_ (1/min)	k_4_ (1/min)	K_FLT_ (mL/min/g)	%ID/g
HCT-116	0.087 ± 0.041	0.094 ± 0.054	0.077 ± 0.013	0.141 ± 0.252	0.042 ± 0.020	8.56 ± 1.17
DiFi	0.105 ± 0.018	0.086 ± 0.019	0.027 ± 0.016	0.027 ± 0.016	0.021 ± 0.005	3.92 ± 1.08
P-value	0.2303	0.4121	0.0061	0.9273	0.0242	< 0.0001

### Adequacy of static [^18^F]-FLT PET to evaluate xenograft tumors

**Figure 4:**
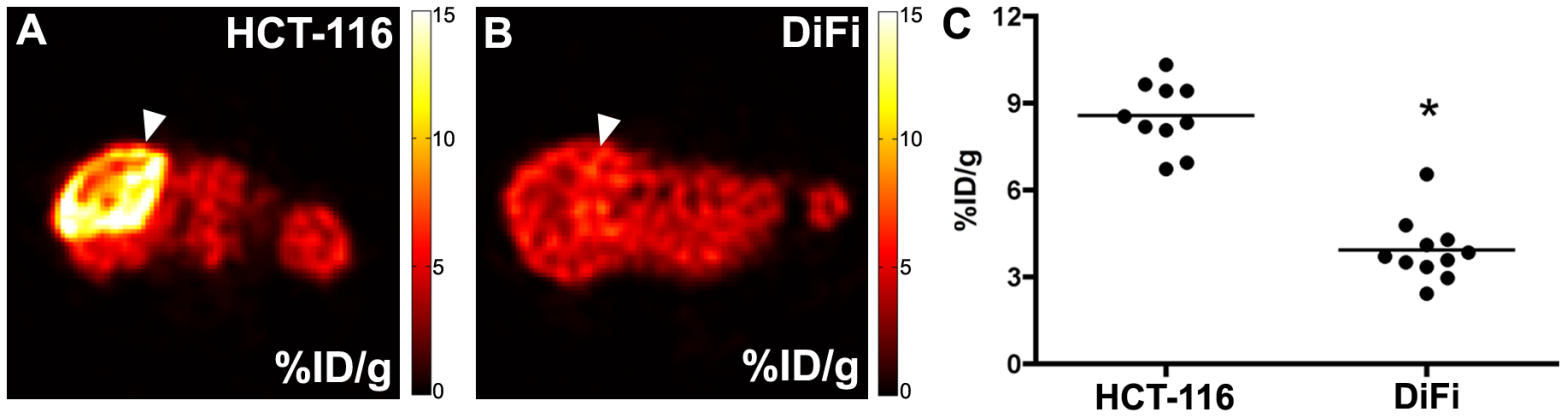
Static [^18^F]-FLT PET uptake is 2-fold greater in HCT-116 xenografts than DiFi xenografts. Representative 20 minute static PET scans reflect the relative 60 minute accumulation of [^18^F]-FLT (%ID/g) qualitatively and show increased tracer uptake in HCT-116 xenografts (A) compared to DiFi (B) xenografts (tumor localized by arrowhead). Spatially, %ID/g maps (A,B) are similar to K_FLT_ maps derived from parametric modeling (**Fig. 3C,F**). Static PET imaging reveals approximately a 2-fold increase in [^18^F]-FLT accumulation in HCT-116 xenografts (8.56 ± 1.17 %ID/g) compared to DiFi xenografts (3.92 ± 1.08 %ID/g; p < 0.0001) (C) and was similar to [^18^F]-FLT flux (K_FLT_) means derived from compartmental modeling (**Table 1**).

Given the complexity and modest throughput of dynamic PET scanning, we evaluated whether similar information regarding [^18^F]-FLT uptake could be gathered from simple, 20 minute static PET scans following a 40 minute tracer uptake phase. Representative images illustrating the relative 60 minute accumulation of [^18^F]-FLT (%ID/g) qualitatively illustrate increased [^18^F]-FLT uptake in HCT-116 xenografts (**Fig. 4A**) compared to DiFi (**Fig. 4B**) xenografts. Similar to flux data derived from dynamic imaging and compartmental modeling (**Table 1**) and TK1 levels by IHC, static imaging resulted in an approximately two-fold difference in [^18^F]-FLT accumulation between HCT-116 xenografts (8.56 ± 1.17 %ID/g) and DiFi xenografts (3.92 ± 1.08 %ID/g) (p < 0.0001). Spatially, the accumulation of [^18^F]-FLT as visualized by static imaging (**Fig. 4A,B**) appeared to be equivalent to K_FLT_ maps derived from compartmental modeling (**Fig. 3C,F**). Given that similar metrics of uptake could be obtained from kinetic modeling of dynamic imaging data (K_FLT_) and simple static imaging (%ID/g) (**Table 1** **,** **Fig. 4C**) , static imaging was used to characterize other cell line xenografts used in the remainder of these studies.

### Effect of de-novo thymidine pathway utilization on [^18^F]-FLT-PET imaging

Using an isogenically matched cell line pair, we initially set out to explore the effect of *p21* deletion upon [^18^F]-FLT-PET, hypothesizing that loss of the cell cycle inhibitor would result in elevated tracer uptake. Indeed, compared to parental HCT-116 cells, HCT-116*p21^-/-^* cells exhibited a significantly greater S-phase fraction as measured by flow cytometry (25.02% vs. 15.31%; p  =  0.0002) (**Fig. S1A**). Interestingly, compared to parental HCT-116 cells, HCT-116*p21^-/-^* cells expressed elevated levels of TS, the enzyme responsible for conversion of deoxyuridine to thymidine, and comparatively diminished levels of TK1 (**Fig. S1B**). We generated parental HCT-116 and HCT-116*p21* ^-/-^ xenograft tumors in nude mice and found that despite this, both models exhibited similar Ki67 indicies (**Fig. S1C**). Thus, parental HCT-116 cells and HCT-116*p21^-/-^* cells appeared to represent an ideal model system to directly evaluate the impact of the *de novo* pathway on [^18^F]-FLT uptake *in vivo*.

**Figure 5:**
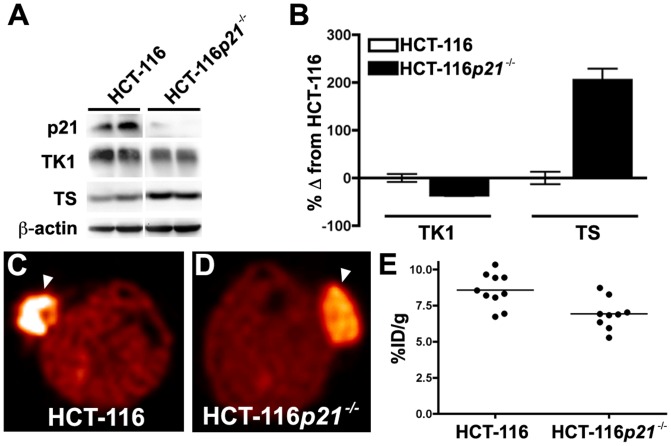
Isogenically matched cell line xenografts illustrate that *de novo* pathway utilization results in decreased [^18^F]-FLT uptake. Western blot analysis illustrated reduced TK1 levels and elevated TS levels in HCT-116*p21* ^-/-^ xenografts compared to wild type HCT-116 xenografts (A). Densitometry of the western blot illustrated that HCT-116*p21^-/-^* tumors exhibited one-third less TK1 protein and double the TS protein compared to the parental line (B). Illustrating the sensitivity of [^18^F]-FLT PET to *de novo* pathway utilization, PET imaging of HCT-116 (C) and HCT-116*p21^-/-^* (D) xenografts closely reflected the relative tumor cell TK1 levels inherent to each model (tumor localized by arrowhead). HCT-116 xenografts (8.56 ± 1.17 %ID/g) exhibited approximately 1/3 greater uptake than analogous HCT-116*p21^-/-^* xenografts (6.91 ± 1.07 %ID/g; p  =  0.005) (E).

Similar to our *in vitro* observations, western blot analysis of HCT-116 and HCT-116*p21* ^-/-^ xenografts illustrated reduced TK1 levels and elevated TS levels in HCT-116*p21* ^-/-^ xenografts compared to the parental cell line (**Fig. 5A**). Western blot densitometry of representative xenografts illustrated that HCT-116*p21^-/-^* tumors exhibited approximately one-third less TK1 protein and approximately double the TS compared to the parental line (**Fig. 5B**). Illustrating the sensitivity of [^18^F]-FLT PET to *de novo* pathway utilization, PET imaging of HCT-116 (**Fig. 5C**) and HCT-116*p21^-/-^* (**Fig. 5D**) xenografts closely reflected the relative tumor cell TK1 levels inherent to each model, where HCT-116 xenografts (8.56 ± 1.17 %ID/g) exhibited approximately 1/3 greater uptake than analogous HCT-116*p21^-/-^* xenografts (6.91 ± 1.07 %ID/g; (p  =  0.005) (**Fig. 5E**). Since others have suggested that endogenous thymidine levels can impact [^18^F]-FLT accumulation in tumors [27], we measured tissue thymidine levels in HCT-116 (37.39 ± 12.61 ng/g tumor) and HCT-116*p21^-/-^* (19.30 ± 11.86 ng/g tumor) xenografts (**Fig. S2**). No statistical difference in thymidine levels was observed (p  =  0.400). These data illustrate that *de novo* pathway utilization results in under-estimation of proliferation by [^18^F]-FLT PET and, importantly, suggests that this metric is incapable of distinguishing moderately proliferative tumors that rely to a greater extent on thymidine salvage from highly proliferative tumors that are more reliant upon the *de novo* pathway.

### Statistical correlation between [^18^F]-FLT-PET and proliferation markers

To more broadly evaluate relationships between [^18^F]-FLT-PET, TK1 levels and Ki67 immunoreactivity in common cell line xenograft models, we systematically evaluated these metrics using a colon-centric panel of human cancer cell lines. The cell lines selected contained representative mutations commonly found in human cancer, including *p53*, *BRAF*, *KRAS*, and *PI3K.* Among nine models, [^18^F]-FLT uptake was highly variable and ranged from 1.66 - 8.89 %ID/g (**Table 2**). The mean uptake across the models was significantly different (Kruskal-Wallis, p <0.0001) . Ki67 positivity was variable amongst the nine cell line models, but to a lesser degree than [^18^F]-FLT uptake (**Table 2**). However, mean Ki67 scoring remained significantly different amongst the cell lines by Kruskal-Wallis analysis (p < 0.0001). TK1 IHC was evaluated in seven of the nine models (**Table 2**); the percentage of TK1 positive cells per field was significantly different amongst those evaluated (p < 0.0001). No statistically significant relationships were observed among mutation status (*p53*, *BRAF*, *KRAS*, *PI3K*) and [^18^F]-FLT uptake, Ki67 IHC scoring, or TK1 IHC scoring. The observed Spearman (nonparametric) correlations between [^18^F]-FLT PET, Ki67, and TK1 are found in **Table 3**. The Spearman correlation coefficient between [^18^F]-FLT uptake and Ki67 indices was -0.07 (p > 0.05), indicating correlation between these metrics consistent with random variation. However, a statistically significant (p < 0.05) Spearman correlation coefficient of 0.36 was observed between [18F]-FLT uptake and TK1 scoring. Among the seven cell line models with both Ki67 and TK1 IHC, a statistically significant (p < 0.05) Spearman correlation coefficient of 0.29 was observed. Using 10,000 bootstrap correlations derived from the data sets, ninety-five percent confidence intervals were constructed from the 2.5^th^ and 97.5^th^ percentiles of the bootstrapped correlations. (**Table 3**). As the ninety-five percent confidence intervals for [^18^F]-FLT uptake and TK1, as well Ki67 and TK1 scoring, did not contain 0, they were considered statistically significant (p < 0.05). However, the correlation between [^18^F]-FLT uptake and Ki67 scoring was not significant.

**Table 2:** Characterization of [^18^F]-FLT uptake, IHC scoring and tumor mutation status in 8 CRC and one breast cancer cell line xenografts.

Cell Line	[^18^F]-FLT uptake (% ID/g)		Ki67 score (% + cells/field)		TK1 score (% + cells/field)		*p53*	*BRAF*	*KRAS*	*PI3K*
	Mean	SD	Mean	SD	Mean	SD				
HCT-116	8.69	1.57	68.50	5.52	38.32	1.90	WT	WT	Mut	Mut
HCT-116*p53^-/-^*	7.77	0.99	64.52	5.56	32.97	4.63	–	WT	Mut	Mut
HCT-116*p21^-/-^*	6.91	1.07	65.08	3.34	19.06	1.71	WT	WT	Mut	Mut
Colo-205	5.65	1.20	62.14	8.99	17.98	4.90	Mut	Mut	WT	WT
Lim 2405	5.24	1.19	85.57	3.63	41.36	4.27	WT	Mut	WT	WT
BT474	4.25	0.79	57.32	4.28	–	–	Mut	WT	WT	Mut
DiFi	3.72	0.99	72.37	12.64	17.95	3.95	Mut	WT	WT	WT
HT-29	3.20	0.65	58.37	9.14	21.73	3.66	Mut	Mut	WT	Mut
SW620	1.66	0.21	74.22	6.35	–	–	Mut	WT	Mut	WT

**Table 3:** Summary of Spearman correlation statistics between [^18^F]-FLT uptake and IHC proliferation markers including 95^th^ percentile confidence intervals from bootstrap estimates.

Marker 1	Marker 2	Spearman Correlation (95% confidence interval)
% ID/g	Ki67	–0.07 (–0.233 to 0.217)
% ID/g	TK1	0.36 (0.142 to 0.643)
Ki67	TK1	0.29 (0.107 to 0.786)

## Discussion

Currently, there is considerable enthusiasm for advancing [^18^F]-FLT PET as a cancer imaging biomarker, especially in drug development. Commercial access to [^18^F]-FLT is increasing, as well as a heightened awareness of the potential of [^18^F]-FLT PET as a non-invasive biomarker in oncology. These efforts should be accompanied by further basic and clinical research to better inform its interpretation and use. [^18^F]-FLT is not a new tracer, and the degree to which [^18^F]-FLT PET quantitatively reflects proliferative index has been continuously debated for more that a decade. Pre-clinical and clinical studies have demonstrated varying degrees of agreement between [^18^F]-FLT uptake in tumors and histological markers of proliferation. In a recent survey of published preclinical studies, [^18^F]-FLT uptake was found to correlate with Ki67 indices in lung cancer [28], B-cell lymphoma [15] and epithelial cancer [28], but did not correlate with Ki67 in colorectal cancer [29], neuroblastoma [30], or across a variety of xenograft types [27]. Clinically, [^18^F]-FLT PET has been correlated with Ki67 to varying degrees as well. Correlation has been observed in lymphoma [31] and thoracic [32], [33] cancers, but not esophogeal tumors [34]. Both correlation and lack thereof has been observed in human lung [16], [35], [36], breast [37], [38], and colorectal [39], [40] cancer. Recently, we found that [^18^F]-FLT PET served as a non-invasive surrogate of Ki67 in Ménétrier's disease, a rare hyperproliferative disorder of the stomach [41]. Unlike our present study, other studies have not always found TK1 levels to correlate with [^18^F]-FLT PET uptake in a number of studies [16], [27]. Based upon this diversity of results and our desire to use non-invasive measurements of proliferation to predict treatment response [21], [22], [41] we explored the relationship between [^18^F]-FLT uptake and cellular metrics of proliferation in a variety of treatment-naive tumors.

Determinants of [^18^F]-FLT PET include delivery, internalization, and intracellular trapping. Delivery may be affected by a number of factors including tissue vascularity, blood vessel permeability, and extravasation. The activity of nucleoside transporters are required to shuttle [^18^F]-FLT from the extracellular to intracellular environment. Via compartmental modeling, we demonstrate that the sum of these processes is similar in two xenograft models that exhibit very large differences in [^18^F]-FLT retention (**Table 1** **,** **Figs. 2** **,** **3**). Even though kinetic modeling results were comparable to static imaging results in simple xenografts, dynamic [^18^F]-FLT PET may still have value in studies where vascularity is uncertain or in certain organ sites. For example, Muzi *et al.* showed that delivery was the dominant factor governing [^18^F]-FLT retention in gliomas where the blood brain barrier has been compromised [13].

Following delivery and internalization in a cell, [^18^F]-FLT is phosphorylated by TK1 to promote trapping which, in turn, generates imaging contrast. TK1 is primarily expressed in late G1- and S-phase of cell cycle before being degraded prior to G2- and M-phase [9]. As such, highly proliferative tumors with comparatively modest fraction of cells in S-phase exhibit similarly modest TK1 levels. This situation was modeled by comparing HCT-116 and DiFi xenografts. In these models, despite similar Ki67 indicies, we observe disparate PCNA and TK1 indicies (**Fig. 2**), which agrees with the difference in [^18^F]-FLT-PET between the models (**Fig. 3** **,** **4**). In this situation, [^18^F]-FLT-PET serves as a much more sensitive marker of DNA synthesis than Ki67, but does not reflect overall proliferation adequately, especially in DiFi xenografts. We showed that utilization of the *de novo* pathway of thymidine synthesis further decouples [^18^F]-FLT-PET from Ki67 indices, as shown in our comparison of the HCT-116 and HCT-116*p21^-/-^* xenografts (**Fig. 5** **)**. These results agree with Moroz *et al.* who suggested that [^18^F]-FLT uptake was unrepresentative of xenograft growth in tumors utilizing the de novo pathway utilization [42]. Recently, Zhang et al. [27] reported that [^18^F]-FLT uptake was inversly related to endogenous thymdine levels. However, in our study which featured the isogenically matched HCT-116 and HCT-116*p21^-/-^* xenografts we failed to observe any relationship between thymidine levels and [^18^F]-FLT uptake (**Fig. S2**).

Perhaps it is unfortunate that Ki67 has historically been the gold standard for validation of [^18^F]-FLT-PET imaging as evidenced by the conflicting correlations between these two markers. Nuclear Ki67 staining is positive for cells in any non-G0 phase of cell cycle and thus serves as a general marker of cell proliferation. In contrast, the closer association of [^18^F]-FLT uptake with TK1 activity, which is typically confined to S-phase, suggests that information regarding proliferation obtained from [^18^F]-FLT-PET imaging is unique and more specific than Ki67. We illustrate this point using 9 different human tumor xenograft cell lines. [^18^F]-FLT uptake is correlated with TK1 indices, but not Ki67 (**Table 3**). Since Ki67 is not S-phase specific, it is reasonable that[^18^F]-FLT-PET may poorly correlate with Ki67, especially in the prognostic setting.

## Conclusion

Our findings illustrate that [^18^F]-FLT-PET reflects tumor proliferation as a function of thymidine salvage pathway utilization. Unlike more generalizable proliferation markers, such as Ki67, [^18^F]-FLT PET reflects proliferative indices to variable and potentially unreliable extents. [^18^F]-FLT-PET cannot discriminate moderately proliferative, thymidine salvage-driven tumors from those of high proliferative index that rely primarily upon *de novo* thymidine synthesis. Accordingly, the magnitude of [^18^F]-FLT uptake should not be considered a surrogate of proliferative index. These may explain, at least in part, the diversity of [^18^F]-FLT-PET correlative results previously reported and suggest future best-practices when [^18^F]-FLT-PET is employed in oncology.

## Supporting Information

Figure S1 ***p21*** **deletion results in elevated S-phase fraction and** ***de novo*** **pathway utilization in HCT-116, although Ki67 remains unchanged.** Compared to parental HCT-116 cells, HCT-116*p21^-/-^* cells exhibited a significantly greater S-phase fraction as measured by flow cytometry (25.02% vs. 15.31%; p  =  0.0002) (A). Compared to HCT-116 cells, HCT-116*p21^-/-^* cells expressed elevated TS protein levels, and comparatively diminished levels of TK1 (B). When grown as xenografts, HCT-116 (68.50 ± 5.52%) and HCT-116*p21^-/-^* (65.08 ± 3.34%; p  =  0.2049) xenografts exhibit similar Ki67 indices (C).
(TIF)

Figure S2 **Endogenous thymidine levels are similar between HCT-116 and HCT-116** ***p21^-/-^*** **xenografts.** HCT-116 tumor xenografts showed similar endogenous thymidine levels (37.39 ± 12.61 ng/g tumor) than HCT-116*p21^-/-^* xenografts (19.30 ± 11.86 ng/g tumor, p  =  0.4000).
(TIF)
