# Supplementary figures and images for: Limits of [18F]-FLT PET as a Biomarker of Proliferation in Oncology
Source: PLoS One. 2013 Mar 15;8(3):e58938. doi: 10.1371/journal.pone.0058938 (PMC3598948; doi:10.1371/journal.pone.0058938)

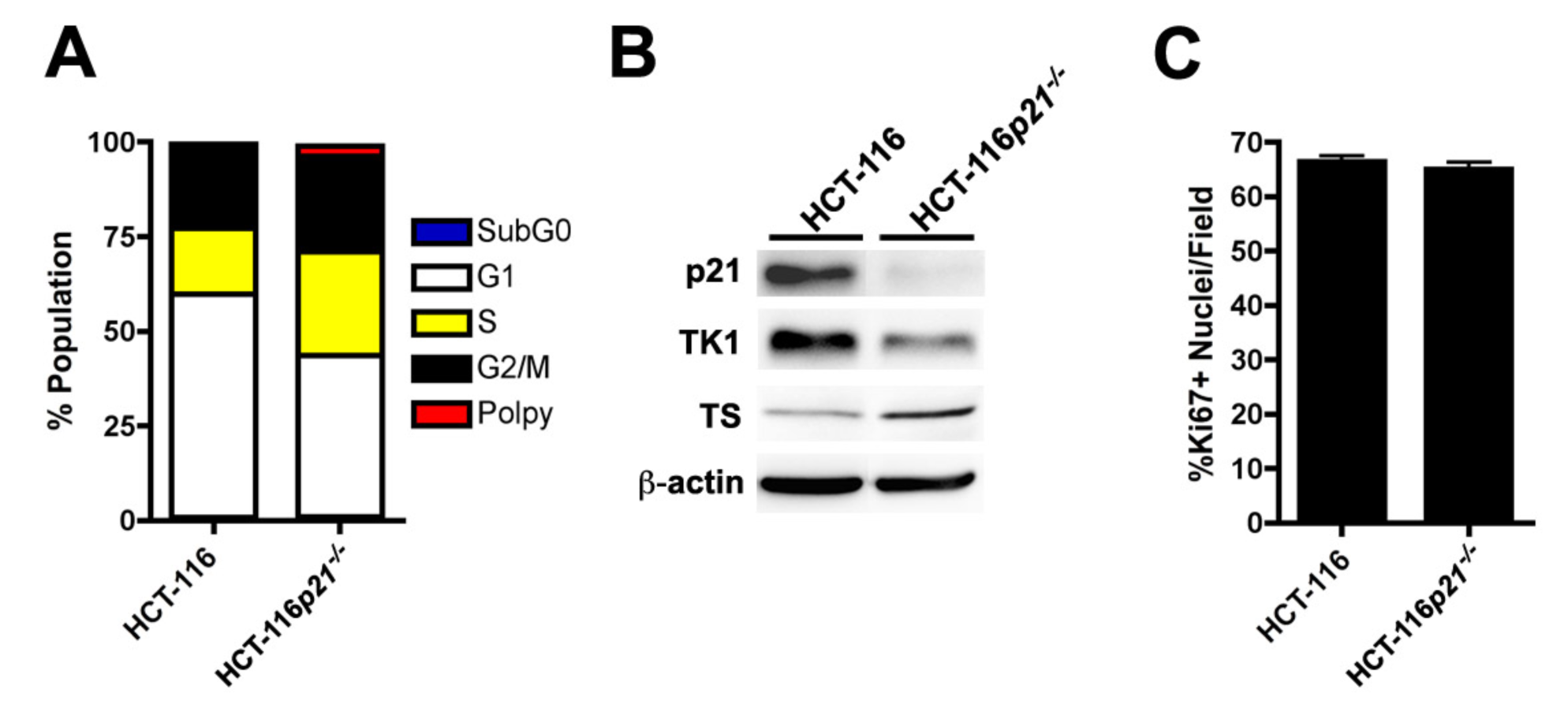

Supplement: Figure S1 — p21 deletion results in elevated S-phase fraction and de novo pathway utilization in HCT-116, although Ki67 remains unchanged. Compared to parental HCT-116 cells, HCT-116p21-/- cells exhibited a significantly greater S-phase fraction as measured by flow cytometry (25.02% vs. 15.31%; p = 0.0002) (A). Compared to HCT-116 cells, HCT-116p21-/- cells expressed elevated TS protein levels, and comparatively diminished levels of TK1 (B). When grown as xenografts, HCT-116 (68.50 ± 5.52%) and HCT-116p21-/- (65.08 ± 3.34%; p = 0.2049) xenografts exhibit similar Ki67 indices (C). (TIF) [file pone.0058938.s001.tif]
